# Relative stability of *Streptococcus mutans* gtfB expression following short-term probiotic exposure in mouth-breathing children: a pilot study

**DOI:** 10.1016/j.jobcr.2026.101490

**Published:** 2026-07-06

**Authors:** Irma Damayanti Suryana, Putri Kusuma Wardani Mahendra, Anrizandy Narwidina

**Affiliations:** a Specialist Study Program of Pediatric Dentistry, Faculty of Dentistry, Universitas Gadjah Mada, Yogyakarta, Indonesia; b Department of Pediatric Dentistry, Faculty of Dentistry, Universitas Gadjah Mada, Yogyakarta, Indonesia

**Keywords:** Mouth-breathing, *Streptococcus mutans*, *gtfB*, Probiotics, *Lactobacillus reuteri*, Gene expression, Pediatric dentistry

## Abstract

**Background:**

Mouth-breathing induces alterations in the oral environment that may promote microbial dysbiosis. *Streptococcus mutans* is a major cariogenic bacterium associated with mouth-breathing conditions, with the *gtfB* gene representing a key virulence determinant involved in bacterial pathogenicity. However, the molecular response of virulence gene expression under altered respiratory conditions remains insufficiently understood.

**Objective:**

This pilot study aimed to investigate the relative stability of *gtfB* gene expression following short-term probiotic exposure in mouth-breathing children.

**Methods:**

A quasi-experimental pre–post study was conducted in a school-based pediatric population**.** Fourteen children aged 9–12 years were recruited, including seven mouth-breathing and seven nasal-breathing controls. Salivary *gtfB* expression was quantified using real-time PCR and normalized to a housekeeping gene**.** Statistical analyses were performed using the Mann–Whitney and Wilcoxon tests.

**Results:**

*gtfB* gene expression was significantly higher in the mouth-breathing group compared with the control group (p < 0.05), indicating increased virulence potential under altered breathing conditions. Following 14 days of *Lactobacillus reuteri* Prodentis administration, a downward trend in *gtfB* expression was observed; however, the change did not reach a statistically significant level (p = 0.398), suggesting relative stability of *gtfB* virulence gene expression.

**Conclusion:**

Short-term probiotic exposure demonstrated limited modulation of *gtfB* expression, indicating relative stability of *Streptococcus mutans* virulence regulation under mouth-breathing–associated dysbiotic conditions.

## 1 Introduction

Mouth-breathing (MB) is recognized as an oral bad habit that may negatively impact oral health. Its prevalence among children aged 6–12 years has been reported to be as high as 59%.^1^ Physiologically, nasal breathing functions to warm, filter, and humidify inhaled air, whereas mouth-breathing results in direct exposure of the anterior teeth and oral mucosa, thereby increasing the risk of oral dryness.^2^ Increasing evidence suggests that mouth-breathing contributes to oral microbial dysbiosis. In children with obstructive sleep apnea (OSA), mouth-breathing is associated with changes in the oral microbiota, particularly an increased abundance of *Streptococcus mutans* and *Lactobacillus* species in saliva.^2^^,^^3^ Mouth-breathing is associated with increased *Streptococcus mutans* level (CFU > 10^5^) and higher plaque index scores.^4^

*Streptococcus mutans* is a major cariogenic bacterium characterized by adhesive capacity, acidogenicity, and tolerance to acidic environments. These virulence traits play a critical role in ecological balance within dental biofilms. An increase in *Streptococcus mutans* promotes the development of a cariogenic biofilm.^5^ *Streptococcus mutans* is commonly used as a biological indicator for caries risk assessment, with salivary levels ranging from 10^5^ to 10^6^ CFU/mL for *Streptococcus mutans* and 10^4^ to 10^5^ CFU/mL for *Lactobacillus* species being associated with an elevated risk of dental caries.^6^

Adhesion of *Streptococcus mutans* is mediated by a sucrose-dependent pathway driven by the activity of *glucosyltransferase* (*gtf)* enzymes. Three gtf genes *gtfB*, *gtfC*, and *gtfD* have been identified in *Streptococcus mutans* and play distinct roles in glucan synthesis.^5^ The *gtfB* gene encodes an enzyme that produces water-insoluble glucans, commonly referred to as *mutans*, consisting of glucose polymers linked by α-1,3 glycosidic bonds. In contrast, *gtfC* is involved in the synthesis of water-soluble glucans (dextrans) with α-1,6 linkages, while *gtfD* primarily contributes to the formation of soluble glucans. Collectively, these glucans enhance bacterial adhesion and aggregation on tooth surfaces and promote biofilm development through interactions with glucan-binding proteins.^7^^,^^8^ Decreased *gtf* activity diminishes the virulence of *Streptococcus mutans*.^5^

The VicRK two-component regulatory system, comprising VicK (sensor histidine kinase) and VicR (response regulator), regulates *gtf* gene expression and plays an essential role in environmental sensing, adaptive regulation, oxidative stress response, acid tolerance, exopolysaccharide (EPS) production, and virulence in *Streptococcus mutans*.^9^, ^10^, ^11^ This regulatory system enables the bacteria to respond to environmental changes such as oxidative stress, pH, acid tolerance, metabolic changes, and exopolysaccharide (EPS) production. The response regulator *VicR* has been demonstrated to bind to the promoter regions of *gtfB*, *gtfC*, and *ftf*, with enhanced regulatory activity observed under high-sucrose conditions concomitant with increased *gtfB* expression.^9^

Probiotic therapy has emerged as a promising strategy for caries prevention by promoting the replacement of pathogenic microorganisms with beneficial commensal bacteria.^12^ The World Health Organization (WHO) defines probiotics as viable microorganisms that provide health benefits to the host when consumed in sufficient quantities.^13^ Probiotics exhibit antimicrobial and antibiofilm activities through the production of various bioactive compounds, including organic acids, hydrogen peroxide (H_2_O_2_), bacteriocins, and surfactants.^14^ Accordingly, this study seeks to assess the impact of *Lactobacillus reuteri* probiotic administration on the expression level of the *gtfB* virulence gene.

## 2 Materials and methods

This study employed a quasi-experimental pre–post design with a control comparison group, conducted in a school-based pediatric population in Yogyakarta, Indonesia. The study was designed to evaluate short-term molecular responses of *Streptococcus mutans* virulence gene expression following probiotic exposure under mouth-breathing conditions. Participants were allocated into two groups: control (n = 7) and experimental (n = 7). Subjects in the experimental group received probiotic lozenges containing *Lactobacillus reuteri* Prodentis (approximately 1 × 10^8^ CFU per lozenge), administered twice daily after tooth brushing in the morning and evening. The total duration of the study was 14 days, including baseline and post-intervention assessments.

### 2.1 Inclusion and exclusion criteria

Ethical approval was obtained from the Institutional Review Board of Universitas Gadjah Mada (No.108/UN1/KEP/FKG-RSGM/EC/2024). Written informed consent was obtained from parents or legal guardians. Participants were children aged 9–12 years with clinically diagnosed mouth-breathing (MB), presenting with DMF-T/def-t index ranging from 0 to 3, and not undergoing orthodontic treatment. Exclusion criteria included antibiotic therapy, probiotic use, or topical fluoride application within one month prior to the study, as well as the presence of systemic disease. Diagnosis of mouth-breathing was established using a multi-step assessment protocol, including Airway Index evaluation, Quinn's Nasal Competency Test, Mallampati classification, tonsillar grading, mirror test, and validated parental questionnaires. Control participants were confirmed as nasal breathers using the same criteria. The probiotic (*Lactobacillus reuteri* Prodentis®, containing approximately 1 × 10^8^ CFU per lozenge) was administered twice daily (morning and night after tooth brushing) for 14 consecutive days. Participants were instructed to allow the lozenge to dissolve slowly in the oral cavity. All participants used the same fluoride-containing toothpaste (Pepsodent®, approximately 1000 ppm fluoride) throughout the study period to standardize oral hygiene conditions. Participants were instructed to maintain consistent dietary habits and to avoid probiotic-containing foods or supplements during the study period. Saliva collection was standardized (morning, at least 1 h after eating or tooth brushing) to minimize variability in salivary flow among subjects. Mallampati classification was assessed according to established airway assessment criteria,^15^ while tonsillar hypertrophy was evaluated using the Brodsky tonsillar grading system.^16^

### 2.2 Sample size calculation

Sample size estimation was performed a priori using G∗Power software (version 3.1.9.7; Heinrich Heine University Düsseldorf, Germany). Based on an assumed large effect size (1.645), a statistical power of 80%, and a significance level of α = 0.01, a minimum of seven participants per group was determined. Given the exploratory nature of this pilot molecular study, the sample size was considered sufficient to detect directional trends in gene expression and to provide preliminary insights into virulence modulation, rather than to establish definitive clinical effects.

### 2.3 Saliva collection, isolation of *Streptococcus mutans*, RNA extraction and qRT-PCR

Quantitative polymerase chain reaction (qPCR) was performed to evaluate *gtfB* gene expression levels of *Streptococcus mutans* in salivary samples obtained from mouth-breathing children and control subjects. Unstimulated saliva (2–3 mL) was collected from each participant between 08:00 and 10:00 a.m. Saliva collection was conducted at the research sites **(**SDN Rejowinangun 1 and SDN Kotagede 1, Yogyakarta, Indonesia**)** under investigator supervision to ensure standardized sampling conditions. RNA was extracted from *Streptococcus mutans* isolated from saliva samples. Bacterial cells were first separated from saliva by centrifugation prior to RNA extraction, ensuring that gene expression analysis was specific to *Streptococcus mutans* rather than whole saliva. All samples were transported to the laboratory within 1 h in an ice box containing gel ice packs to ensure temperature preservation. qPCR analysis was performed at two time points: prior to and following 14 days of probiotic intervention. Saliva samples were collected at least 1 h after the subjects had brushed their teeth or eaten.

The qPCR program consisted of an initial denaturation at 95°C for 5 min, followed by 38 cycles of 30 s at 95°C, 30 s at 49°C, and 45 s at 72°C, with a final extension step at 75°C for 5 min. Amplification of the *gtfB* gene was performed using gene-specific primers listed in Table 1. Gene expression levels were normalized to *lepA*, which served as the housekeeping gene. Total RNA from *Streptococcus mutans* in saliva samples was isolated using a commercial RNA extraction kit according to the manufacturer's protocol. RNA quality and concentration were assessed prior to downstream analysis.

**Table 1:** Gene-specific primers.

No	Gene Target	Gene candidate	*Basepair*
1.	*Glucosyltransferase/*GtfB (*Streptococcus mutans)*	**Candidate 1** AGCAATGCAGCCATCT ACAAAT **F** ACGAACTTTGCCGTT ATTGTCA **R** **Candidate 2** ACTACACTTTCGGGTGGCTTGG **F** CAGTATAAGCGCCAGTTTCATC **R**	98 bp
2.	*LepA*	CTCTATTATTGCCC **F** TACATCACCCGTTG **R**	441 bp

### 2.4 Statistical analysis

Statistical analysis was performed using SPSS version 27 (IBM Corp., Armonk, NY, USA). Data normality was assessed using the *Shapiro–Wilk* test, and homogeneity of variance was evaluated using *Levene's* test. Between-group comparisons were analyzed using the *Mann–Whitney* test, while within-group comparisons were assessed using the *Wilcoxon* signed-rank test. Statistical significance was set at p < 0.05.

## 3 Results

The assessment of Mallampati classification and tonsillar size indicated that Grade 2 (The uvula, hard palate, and soft palate were visible)^15^ was the most prevalent Mallampati score in the mouth-breathing group (n = 4), with a similar distribution observed in the control group. In terms of tonsillar grading, most mouth-breathing subjects exhibited Grade 3 hypertrophy (Tonsils occupied 50–75% of the oropharyngeal space).^16^ A comparable pattern was observed in the control group, where Grade 3 was also the predominant finding (Table 2). Diagnostic parameters used to classify mouth-breathing and nasal-breathing subjects, including Mallampati classification and tonsillar grading, are presented in Table 2.

**Table 2:** Mallampati and tonsillar examination.

Group	Mallampati score				Tonsil score			
	Grade 1	Grade 2	Grade 3	Grade 4	Grade 1	Grade 2	Grade 3	Grade 4
Mouth breather children	2	4	1		1		4	2
Control	3	4			4	2	1	

**Fig. 1:**
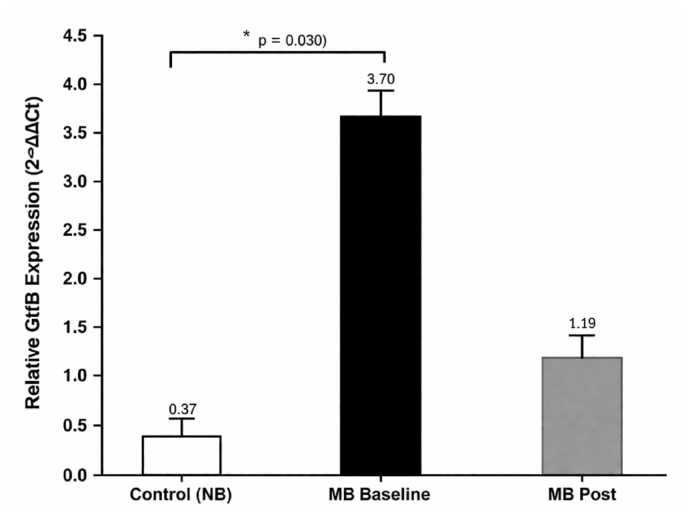
Relative expression of the *gtfB* gene (2^-ΔΔCt) in nasal-breathing controls and mouth-breathing children at baseline and after 14-day probiotic intervention.

The relative expression of the *gtfB* virulence gene was quantified using real-time PCR and expressed as 2^-ΔΔCt values. Measurements were obtained from nasal-breathing controls and mouth-breathing children at baseline and after a 14-day probiotic intervention (Table 3; Fig. 1).

**Table 3:** Relative expression of the *gtfB* gene (2^-ΔΔCt) in study groups.

Group	Baseline 2^-ΔΔCT	Post-intervention 2^-ΔΔCT	p Value
Nose-breather children (control)	0.37	-	0.030^a^
Mouth-breather children	3.70	1.19	0.398^b^

a Between-group comparison at baseline (*Mann–Whitney* test).

b Within-group comparison (baseline vs post-intervention; *Wilcoxon* signed-rank test).

A comparison of the mean 2^−^ΔΔCT values between the control group and the mouth-breathing group at baseline was analyzed using the *Mann–Whitney* test, yielding a statistically significant result (p = 0.03; p < 0.05). In contrast, within the mouth-breathing group, comparison of baseline and post-treatment values after 14 days of probiotic therapy was analyzed using the *Wilcoxon* and showed no statistically significant change (p = 0.398; p > 0.05). A reduction in *gtfB* gene expression was observed following probiotic administration (from 3.702 to 1.19), although the change did not reach statistical significance. These findings indicate a relative stability of *gtfB* gene expression despite short-term probiotic exposure, suggesting that virulence regulation may be maintained under mouth-breathing–associated conditions.

## 4 Discussion

### *4.*1 gtfB expression levels in mouth- and nasal-breathing children

The findings of this study indicate that *gtfB* gene expression was elevated in mouth-breathing children compared with their nasal-breathing counterparts, underscoring the influence of respiratory patterns on oral ecological balance and dental health. Prolonged mouth-breathing is associated with oral mucosal dehydration, impaired salivary clearance, gingival inflammation, and cariogenic biofilm formation.^4^^,^^17^^,^^18^

Increased *gtfB* expression promotes bacterial adhesion, enhances biofilm stability, and augments the resistance of *Streptococcus mutans* to mechanical disruption and host immune responses. The expression of this gene is tightly regulated by two-component signal transduction system comprising the VicK sensor (histidine kinase) and the VicR (response regulator), which enables *Streptococcus mutans* to sense and adapt to environmental cues through coordinated modulation of virulence-related genes.^10^^,^^19^^,^^20^ This regulatory adaptability may contribute to the persistence of virulence gene expression under altered environmental conditions, including mouth-breathing.

These observations are consistent with previous reports demonstrating that mouth-breathing children exhibit lower salivary pH, elevated intraoral temperature, and reduced salivary flow rates compared with nasal breathers.^17^ Such alterations compromise the self-cleansing function of saliva, thereby facilitating microbial dysbiosis. Accordingly, higher *Streptococcus mutans* counts and increased plaque index scores have been consistently reported in this population (Fan et al., 2020; Mummolo et al., 2024).^2^^,^^18^ Collectively, these findings support the notion that mouth-breathing creates a microenvironment conducive to sustained virulence gene expression.^2^^,^^4^^,^^17^^,^^18^

### 4.2 Molecular characterization and genomic organization of *gtfB* in mouth-breathing children

The *gtfB* promoter region harbors specific binding motifs for the response regulator *VicR* following activation of the VicRK two-component system, which is triggered by environmental stimuli such as pH variation, oxidative stress, and nutrient limitation.^11^ Bioinformatic analyses have identified conserved −10 and −35 elements corresponding to RNA polymerase binding sites, along with VicR operator sequences that confer environmental stress responsiveness to *gtfB* expression, thereby facilitating the synthesis of water-insoluble glucans and the formation of cariogenic biofilms.^21^

Regulation of *gtfB* is orchestrated through the VicRK signaling pathway, in which the sensor histidine kinase *VicK* detects environmental stress signals and undergoes autophosphorylation, followed by phosphate transfer to the response regulator VicR. The phosphorylated VicR subsequently binds to the *gtfB* promoter, activating transcription and enhancing GtfB enzyme production, which in turn promotes insoluble glucan synthesis, biofilm maturation, and increased bacterial resistance to host immune defenses and mechanical removal ^19^^,^^22^.Such multi-layered regulatory control suggests that *gtfB* expression may exhibit apparent stability in response to short-term environmental or therapeutic perturbations, particularly under adaptive stress-response signaling conditions ^11^^,^^21^^,^^22^.

Alterations in this regulatory axis, including promoter reorganization, mutational changes, or modulation of intron-like regulatory elements within the *gtfB* locus, may disrupt signal transduction and attenuate the biofilm-forming capacity and virulence of *Streptococcus mutans* ^23^.

### 4.3 Effect of *Lactobacillus reuteri* probiotic administration in mouth-breathing patients

Administration of *Lactobacillus reuteri* probiotics resulted in a reduction in *gtfB* gene expression levels in mouth-breathing children, although the decrease did not reach statistical significance. Despite this non-significant change, a downward trend was observed, indicating a limited modulation rather than absence of biological response.

This finding is consistent with previous studies by Alamoudi et al. (2018),^12^ which reported a reduction in salivary *Streptococcus mutans* counts following *Lactobacillus reuteri* supplementation in children, as well as by Noda et al. (2021),^24^ who demonstrated downregulation of *gtfB* expression after probiotic exposure. The *gtfB* gene plays a pivotal role in the synthesis of water-insoluble glucans, which are essential for bacterial aggregation and plaque formation on tooth surfaces.

The observed reduction in *gtfB* expression may be attributed to the antimicrobial mechanisms exhibited by *Lactobacillus reuteri*. Several potential mechanisms have been proposed, including bacteriocin release, co-aggregation with pathogenic bacteria, and competitive inhibition of cariogenic microorganisms for nutrient availability and adhesion sites. Notably, *Lactobacillus reuteri* possesses unique metabolic capabilities, such as the production of hydrogen peroxide (H_2_O_2_) and reuterin via the glycerol fermentation pathway, both of which can compromise the survival and virulence of *Streptococcus mutans*. Reuterin, in particular, is recognized as a broad-spectrum antimicrobial agent with several advantages over conventional antimicrobials.^25^^,^^26^ Its antibacterial activity involves the disruption of free sulfhydryl groups in bacterial proteins and the induction of intracellular oxidative stress.

Supporting this mechanism, Zhang et al. (2021)^19^ reported downregulation of *Streptococcus mutans* virulence genes, including *gtfB*, following 24 h of reuterin exposure. In addition, *Lactobacillus reuteri* derived H_2_O_2_ exhibits cytotoxic effects on bacterial cells by damaging nucleic acids, proteins, and lipid membranes. Given the critical role of nucleic acids in gene regulatory networks, such cytotoxic stress may interfere with the regulation of virulence gene expression.^27^ Dysregulation of *gtf* genes, including *gtfB*, can impair the biofilm-forming phenotype of *Streptococcus mutans*.^24^ This observation is supported by Fitri et al. (2025),^20^ who reported that deletion of *gtfB* and *gtfC* significantly reduces biofilm formation and polysaccharide synthesis. However, *Streptococcus mutans* possesses adaptive defense mechanisms against antimicrobial stress mediated by two-component regulatory systems (TCS). Matsuo and Komatsuzawa (2017) demonstrated that TCS play a critical role in *Streptococcus mutans* resistance to bacterial byproducts, including H_2_O_2_.^22^ Among these systems, VicRK is closely associated with resistance to oxidative stress induced by H_2_O_2_ exposure. This adaptive response may contribute to the relative stability of gtfB expression observed in the present study despite probiotic exposure. These findings suggest that virulence gene regulation in *Streptococcus mutans* is not solely dependent on external antimicrobial pressure but is also governed by intrinsic adaptive regulatory mechanisms.^21,22^

This adaptive response may partially explain the lack of statistically significant changes observed in the present study, despite a downward trend in *gtfB* expression following *Lactobacillus reuteri* administration. These findings indicate that *Lactobacillus reuteri* probiotics may modulate *gtfB* gene expression in mouth-breathing children; however, host microbe interactions and bacterial stress-response mechanisms may constrain the extent of this effect.^21^^,^^22^ The small sample size reflects the pilot nature of this molecular study and was intended to detect large effect trends rather than definitive clinical efficacy**.** In addition, the present study was designed as a pilot quasi-experimental molecular investigation aimed at exploring short-term trends in virulence gene expression rather than evaluating clinical efficacy outcomes. Therefore, the study protocol was not prospectively registered in a clinical trial registry platform, which should be acknowledged as a methodological limitation. Future confirmatory studies involving larger samples and clinical outcome measures should incorporate prospective trial registration. Accordingly, the observed stability of gene expression should be interpreted as a preliminary indication of microbial resilience rather than absence of probiotic activity.^22^^,^^24^, ^25^, ^26^.

## 5 Conclusion

1. *gtfB* gene expression was significantly higher in mouth-breathing children compared to nasal breathers (3.70 vs 0.37; p = 0.03).
2. After 14 days of *Lactobacillus reuteri* administration, gtfB expression decreased (3.70 to 1.19), but the change was not statistically significant (p = 0.398).
3. These findings indicate relative stability of *gtfB*-mediated virulence following short-term probiotic exposure.
4. Short-term probiotic intervention may be insufficient to significantly modulate virulence gene expression under mouth-breathing conditions.

## Patient's/guardian's consent statement

Written informed consent was obtained from all parents or legal guardians prior to participation*,* ensuring full understanding and voluntary participation**.**

## Ethical clearance statement

Ethical approval was granted by the Institutional Review Board of Faculty of Dentistry, Universitas Gadjah Mada and RSGM UGM Prof. Soedomo (No.108/UN1/KEP/FKG-RSGM/EC/2024). Parental consent and child assent were obtained prior to enrollment.

## Funding

The authors acknowledge the support of the Community Research Grant Fund of the 10.13039/501100023915Faculty of Dentistry, 10.13039/501100012521Universitas Gadjah Mada (UGM) (No.3918/UN1/KG/Set.KG1/LT/2024).

## Declaration of competing interest

The authors declare that they have no known competing financial interests or personal relationships that could have appeared to influence the work reported in this paper.
